# The acute lymphoblastic leukemia prognostic scoring whether it is possible by BCL-2, BAX gene promoter genotyping

**Published:** 2016

**Authors:** Mozhagan Moazami-Goudarzi, Majid Farshdousti-Hagh, Abbasali Hoseinpour-Feizi, Mehdi Talebi, Ali Akbar Movassaghpour-Akbari, Karim Shams-Asanjan, Jamal Eyvazi-Ziyaee, Morteza Seifi

**Affiliations:** 1 Stem Cell Research Center, Tabriz University of Medical Science, Tabriz, Iran.; 2 Hematology and Oncology Research Center, Tabriz University of Medical Science, Tabriz, Iran.; 3 Department of Medical Genetics, University of Alberta, Alberta, Canada

**Keywords:** Acute lymphoblastic Leukemia, BAX, BCL2, Prognostic scoring, Survival, Single Nucleotide Polymorphism.

## Abstract

**Background:**

BCL-2 is the most important anti-apoptotic regulator and Bax is a pro-apoptotic protein. The status of these parameters or the ration of BCL-2 to BAX is important in malignant cell fate as well as normal cells.

**Methods:**

Sixty-two ALL patients and 62 healthy sex-and age-matched controls were studied. After genotyping, the promoter region of the BAX and BCL-2 genes by RFLP-PCR method the patients were classified in nine prognostic groups, after that, the overall survival ratio of each score was compared with others pair-wise or between groups.

**Results:**

The frequencies of the AA, AC, CC alleles of the BCL-2 C-938A polymorphism in patient group were 33 (53.23%), 18 (29.03%), 11 (17.74%), and in the control group were 13 (21.0%), 27 (43.5%), 22 (35.5%), respectively (P=0.003). Also, the frequencies of AA, AG, GG alleles of the BAX G-248A SNP were 15 (24.2%), 24 (38.7%), 23 (37.1%) in ALL group and 13 (21.0%), 25 (40.3%), 24 (38.7%) (p>0.05) in the control group. The survival time estimation and ratio were significantly different between different SNPs in BCL-2 (P=0.002).

**Conclusion:**

These findings showed that the BCL-2 promoter region polymorphism is more reliable than BAX gene promoter polymorphism in any ALL scoring system. But the establishment of complete scoring system requires further more clinical and laboratory findings along with genetic polymorphisms is necessary.

Fully controlled cell turnover guarantees tissue homeostasis which both proliferation and apoptosis are important in maintaining this balance (1). Apoptosis begins through two different pathways: cells may receive intrinsic death stimuli such as excessive oncogene activation, DNA damage, unfolded protein response, or they may respond to extrinsic death stimuli such as binding of Fas or TNFα ligands to their receptors on the cell surface as part of the effector phase of an immune response. These pathways converge on the mitochondrial outer membrane, where the BCL2 protein family plays a pivotal role in the regulation of apoptosis (2). This family is one of the key regulators of balance between proliferation and apoptosis, that is divided to pro-apoptotic members such as BCL2-associated X protein (BAX) and BAX-like death factor (BAK) and anti-apoptotic members such as BCL2 itself and BCL2-like 1 (Bcl-xL), which protects cells from apoptosis (3). It has been shown that high *BCL2* expression is associated with poor survival in patients with malignancies such as prostate cancer (4).

The *BCL2* gene is located on chromosome 18q21.3 and consists of three exons and two promoter regions, each with different functional properties. The second promoter (P2) is located 1.4 Kbp upstream from the translation initiation site, and its activation decreases P1 activity. It thus functions as a negative regulatory element (5, 6). Nückel et al. showed that there is a significant relationship between the C allele of the C-938A (rs2279115) single nucleotide polymorphism (SNP) and P2 activation, possibly, because the increased interaction between the P2 promoter region and transcription factors results in lower expression of the BCL2 protein (7).

The BAX protein is produced by a tumor suppressor gene that belongs to the BCL2 family. It releases cytochrome by producing channels in the outer mitochondrial membrane of (8), and thereby leads to caspase activation. BAX molecules oligomerize and interact with other BAX and other members of the BCL2 family, and this in turn influences protein activity (9). The *BAX* gene is located in chromosome 19. It has been reported that the *BAX* G-248A (rs4645878) SNP polymorphism affects *BAX* expression, with higher levels of expression associated with the GG allele than the GA or AA alleles (10).

The accurate staging of leukemia is a crucial factor in treatment efficacy. Almost all cancer staging systems are based on the size and spread of tumors, but because leukemia develops in growing cells of the peripheral blood and bone marrow, its staging is different. In general, the stages of leukemia are based on blood cell counts and the infiltration of other organs such as the liver and spleen by leukemia cells. We developed a new system to classify the prognosis for ALL based on the promoter region SNPs present in the genes of the two key pro- and anti-apoptotic pathway regulators, the *BAX* and *BCL2* genes.

## Methods

The study was designed as case-control method, and patients were selected stochastically during over six month period. Each patient at any stage of disease was followed-up for an additional one year. Patients who did not complete the study were excluded.

**Participants: **The participants were 62 patients with ALL diagnosed on the basis of hematopathological criteria. All patients were seen by a pediatric oncologist at the Hematology Oncology Department of Tabriz Children’s Hospital. During inclusion, the participants were at various stages of the anti-leukemia therapy (i.e. remission induction, maintenance therapy, post-therapy follow-up). The time elapsed from the onset of disease ranged from new cases to patients with 10 years duration of follow-up period. All patients underwent morphological characteristics of the bone marrow as well as flow-cytometery to evaluate the expression of CD10, CD19, HLA-DR, CD20, CD22, CD3, CD34 and CD45 have been prepared based on protocols for routine hospital practice. Clinical and hematological data were extracted from the patient’s files or from direct examination and interview with the participant or relatives. The control group consisted of 62 sex- and age-matched healthy children who were selected from the population of children who received a routine pre-school physical examination; none of the children in the control group had any known disease at the time of examination. All phases of the study were carried out according to the Declaration of Helsinki, and the study was approved by the Tabriz University of Medical Sciences Ethics Committee.

Samples of peripheral blood (2-5 mL depending on body weight) were drawn and stored at −80°C until testing. Based on previous studies of the role of *BCL2* and *BAX* polymorphisms on the expression levels of related proteins (7), we designed a classification system consisting of 9 groups based on *BCL2* and *BAX* gene SNPs as shown in table I, and each patient was followed for 1 additional year.

**DNA extraction: **DNA from whole blood cells was extracted with a salting out method. DNA concentration and purity in each sample were measured with a NanoDrop 1000 Thermo scientific Spectrophotometer (Wilmington, DE, USA). DNA extracts with an optical density ratio between 1.6 to 1.9 at 260/280 nm were chosen for the subsequent steps. Extracted DNA samples were stored at −70°C until testing. 

**Amplification of the BCL2 promoter region: **To determine the *BCL2 *genotype in samples, genomic DNA was amplified with the forward primer: 5´-TTATCCAGCTTTTCGG-3´ and the reverse primer, 5´-GGCGGCAGATGAATTACAA-3´. Primers were prepared by Bioneer (Daejeon, S. Korea). Polymerase chain reactions (PCR) were performed with a SensoQuest Thermocycler (Göttingen, Germany) in a final volume of 25 µL, containing 12.5 µL Master Mix Red (Ampliqon, Odense, Denmark), 1.25 µL of each primer, 6 µL dH_2_O and 4 µL genomic DNA. The PCR amplification cycles consisted of initial denaturation at 94°C for 5 min followed by 35 cycles of 30 s at 94°C, 30 s at 52°C and 30 s at 72°C, with a final extension cycle of 10 min at 72°C.

**Figure 1:**
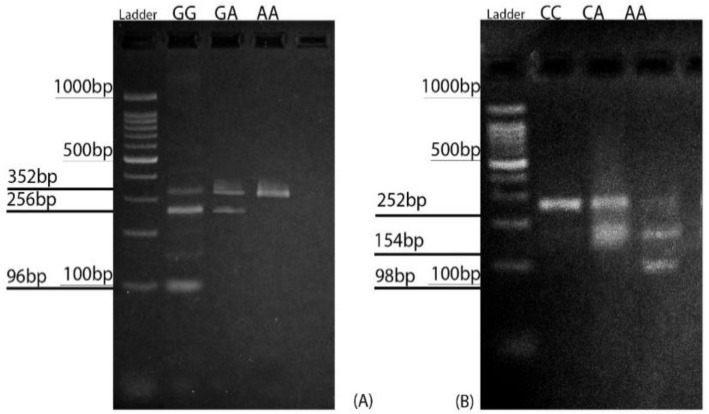
Restriction fragment length polymorphisms in BAX G-248A (rs4645878) and BCL2 C-938A (rs2279115). (A) The BAX promoter allele GG produced three restriction fragments: 352 bp, 256 bp and 96 bp. The GA allele produced 352-bp and 256-bp fragments; the 96-bp band was usually invisible. The AA allele produced a single 352-bp fragment. (B) The CC allele of the BCL2 promoter region produced a single 252-bp fragment, the CA allele produced 252-bp and 154-bp fragments, and the AA allele produced 154-bp and 98-bp fragments

**Restriction enzyme analysis of BCL2 gene: **The restriction enzyme BccI (New England BioLabs, Ipswich, UK ) was used to screen the samples for the C-938A (rs2279115) SNPs. Aliquots of 6 µL of each PCR product were digested with 1 unit of restriction enzyme at 37°C overnight together with 1 µL 10 enzyme buffer. Aliquots of 4 µL of the digested PCR products were then electrophoresed in 1.5% agarose gel at 110 V for 40 min in an electrophoresis apparatus. The PCR products were visualized after staining with safe stain (Sinnagene, Tehran, Iran). The homozygous CC (wild-type) genotype was visualized as a single major 252-base pair (bp) band. The AC heterozygous genotype showed both the undigested 252-bp band and the digested 154- and 98-bp bands, and the fully digested 154- and 98-bp products represented the AA genotype (figure 1A).

**Amplification of the BAX promoter region: **To determine the *BAX* genotype of the samples, genomic DNA was amplified with the forward primers 5′-CGGGGTTATCTCTTGGGC-3′ and the reverse primer 5′-GTGAGAGCCCCGCTGAAC-3′. PCR was performed in a final volume of 25 µL containing 12.5 µL Master Mix Red (Ampliqon), 1.25 µL of each primer (Bioneer), 6 µL dH_2_O and 4 µL genomic DNA. The PCR cycles consisted of initial denaturation at 95°C for 5 min followed by 30 cycles for 30 s at 94°C, 30 s at 64.3°C and 45 s at 72°C, with a final extension cycle of 5 min at 72°C.

**Restriction enzyme analysis of the BAX gene: **The restriction enzyme AciI (New England BioLabs) was used to screen samples for the *BAX* G-248A (rs4645878)SNP. The PCR products (6 µL) were incubated overnight with 1 µL restriction enzyme at 37°C. Aliquots of 4 µL of the digested PCR products were then electrophoresed on a 2% agarose gel at 150 V for 50 min in an electrophoresis apparatus. The PCR products were visualized after staining with safe stain (Sinnagene). 

The homozygous GG (wild-type) genotype was visualized as three major bands of 352, 256, and 96 bp. It should be noted that the 256-bp band was the most intense. The heterozygous AG genotype resulted in the loss of a restriction site for AciI in one of the *BAX *promoters, and showed three bands, with the 352-bp band being the most intense, whereas the 96-bp band became almost invisible. The homozygous AA genotype (homozygous carrier of the SNP) showed only the 352-bp band (figure 1B).

**Statistical analysis: **Descriptive statistics were compiled and reported as the mean ± standard deviation. Comparison of the means between parametric data for two independent groups was done with *t*-tests for independent samples. For nonparametric data and data that were not normally distributed, Mann-Whitney U test, Kruskal-Wallis test and chi-square test were used. Overall survival ratios were estimated with the Kaplan-Meier method. All statistical analyses were done with IBM-SPSS Version. 22 software (Chicago, IL, USA) and p values <0.05 were regarded as significant. 

## Results

The mean age (±SD) of patient was 7.4±3.37 years, and in the control group was 7.18±3.84 years (P=0.729). Among patients with ALL, 41(66.1%) were boys and 21(33.9%) were girls. In the control group, 36 participants (58.1%) were boys and 26 (41.9%) were girls (p>0.05). In the ALL group, 50% of the patients were new cases, 36% in follow-up and 14% in relapse phase. Table 1 shows the frequencies and percentages of patients in each scoring category based on our classification system.

**Table 1:** The scoring system to predict the prognosis in patients with acute lymphoblastic leukemia, based on previous studies of BAX and BCL2 promoter region polymorphisms and expression levels

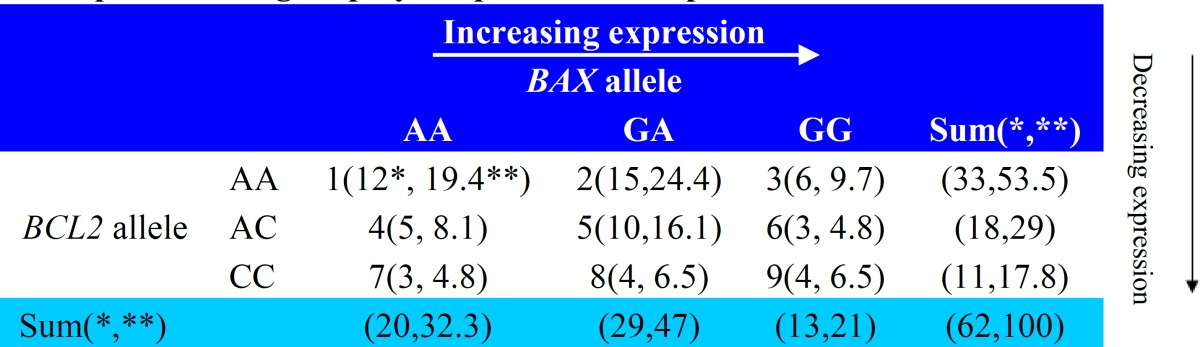

White blood cell count (mean±SD) was 4.5±3.610^9^/L in the patient group ranging from 1.310^9^/L-2110^9^/L and 7.18±1.810^9^/L in the control group (p<0.05) ranging from 3.410^9^/L to 11.4010^9^/L. Genotyping of the promoter region of the *BCL2* gene (C-938A) showed the following allele frequencies in the ALL group: AA in 33 children (53.23%), AC in 18 (29.03%) and CC in 11 (17.74%). The corresponding frequencies in the control group were AA in 13 children (21.0%), AC in 27 (43.5%) and CC in 22 (35.5%). The polymorphism frequencies differed significantly between groups (P=0.003) (table 1).

**Figure 2:**
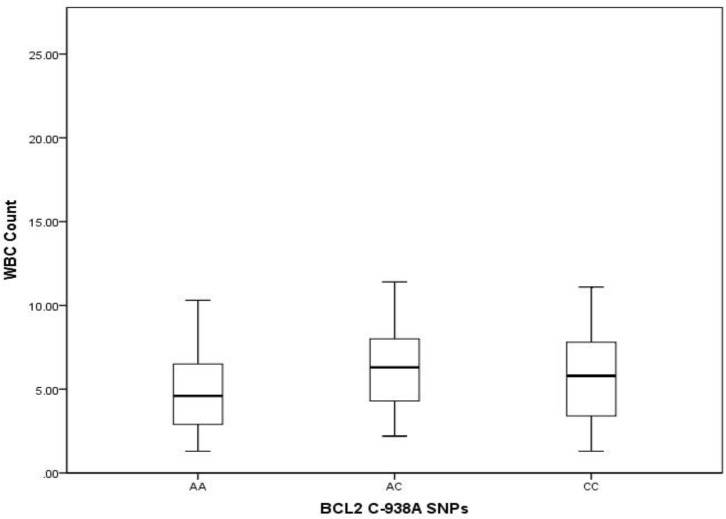
Distribution of white blood cell counts and *BCL2* SNPs (P=0.032

**Figure 3:**
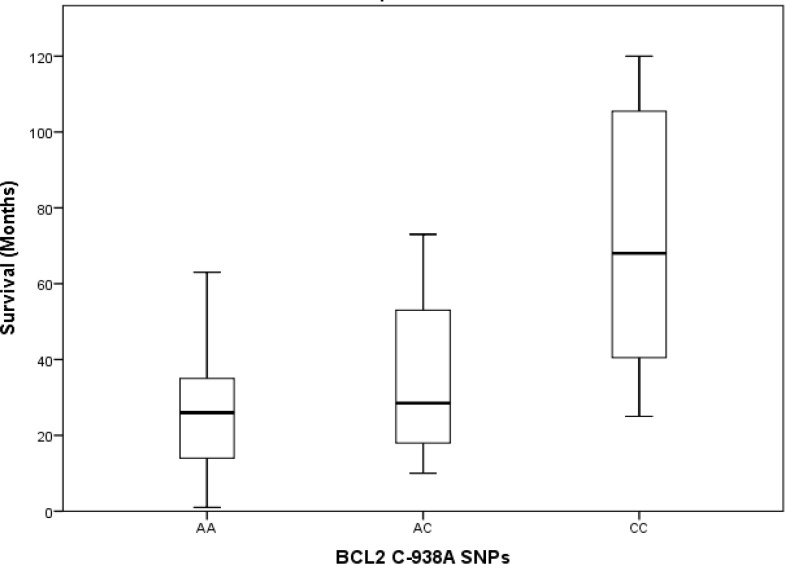
Distribution of survival times among *BCL2* gene SNPs in patients with acute lymphoblastic leukemia (P=0.041

Median survival times in patients with different genotypes were AA allel, 26 months, AC allel, 29 months and CC, 68 months. By comparing the medians of independent samples we found that median white blood cell (WBC) count varied across categories of *BCL2* polymorphism (P=0.032) (figure 2), and comparisons of the distribution of WBC counts across *BCL2* allele polymorphisms with the Kruskal-Wallis method showed a statistically significant trend in WBC count distribution (P=0.041) (figure 3). 

**Figure 4:**
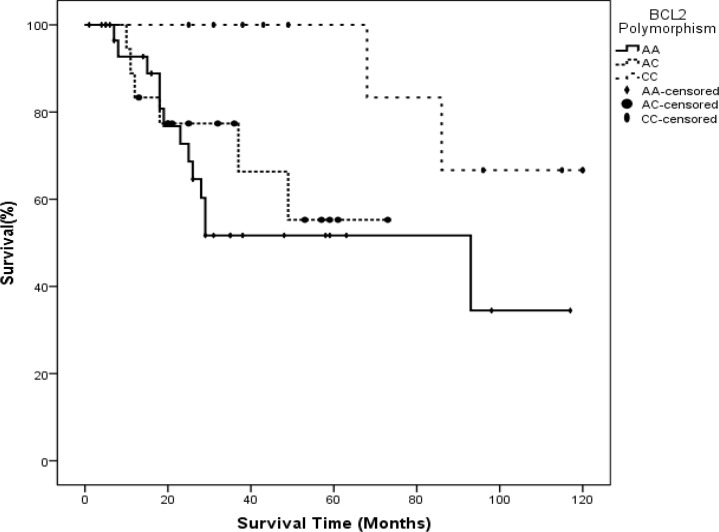
Kaplan-Meier log-rank test for survival time estimates in patients with different *BCL2* SNPs

However, we found no statistically significant differences between median age and age distribution in patients with different *BCL2* C-938A polymorphisms (p>0.05) according to the Kruskal-Wallis test. The distribution of survival times across types of *BCL2* promoter region polymorphisms differed significantly (P=0.002) (figure 4). 

**Figure 5:**
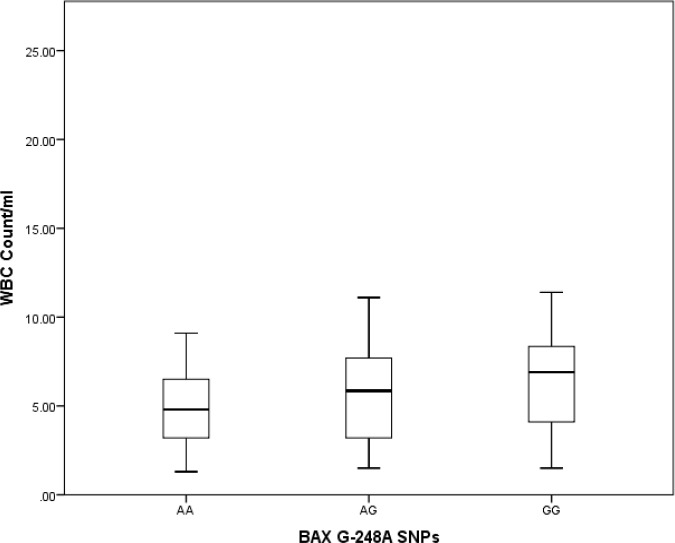
Distribution of white blood cell counts in patients with different *BAX* gene SNPs (p>0.05).

**Figure 6:**
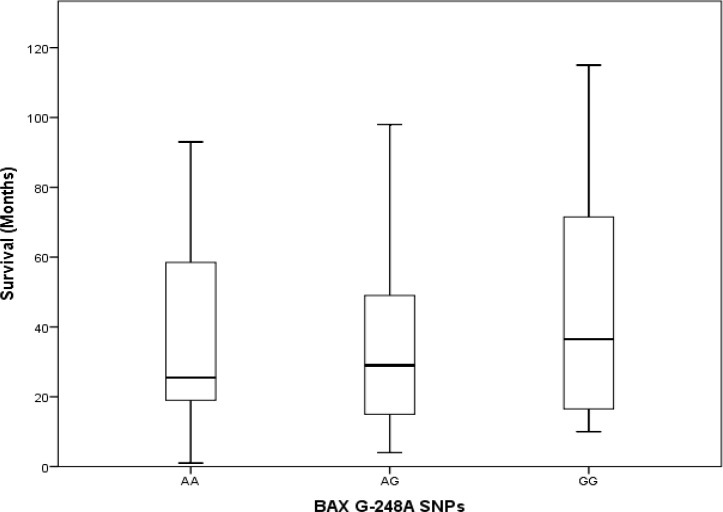
Distribution of survival times in patients with acute lymphoblastic leukemia and different *BAX* gene SNPs (p>0.05).

**Figure 7:**
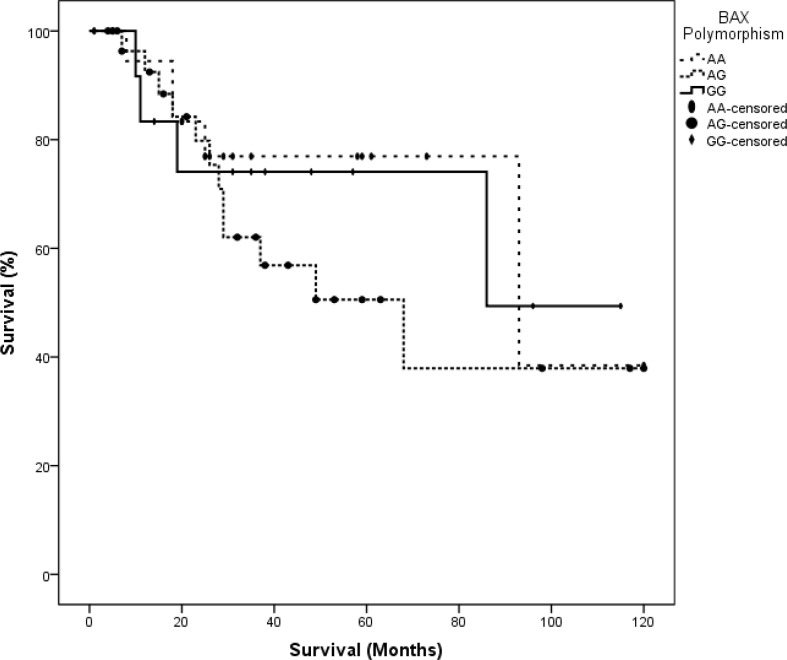
Kaplan-Meier log-rank test for survival time estimates patients with different *BAX* SNPs (P=0.592).

In the control group, there was no statistically significant difference in the distribution of age or WBC count. Kaplan-Meier tests to estimate survival time yielded 66.39±9.56 months for the AA allele, 52.76±6.56 months for the AC allele and 105.67±8.54 months for the CC allele. Pairwise comparisons of survival times associated with different alleles showed statistically significant differences between AA vs. CC (P= 0.025) and AC vs. CC (P=0.040), but no significant difference between AA vs. AC (P=0.587) (Figure 4). Genotyping the *BAX* gene untranslated G-248A region showed that in patients with ALL, the SNP frequencies were GG in 23 (37.1%), GA in 24 (38.7%) and AA in 15 (24.2%). In the control group, the corresponding frequencies were GG in 24 (38.7%), GA in 25 (40.3%) and AA13 (21.0%). We found no statistically significant differences between polymorphism frequencies between the ALL and control groups (table 1). Mean survival times associated with different alleles were GG, 37 months, AG, 29 months and AA, 26 months. Kruskal-Wallis tests to compare the distributions of age, WBC count and survival time (ALL group only) in our two groups detected no statistically significant differences between types of *BAX* G-248A polymorphisms (p>0.05). Kaplan-Meier tests to estimate survival times associated with different *BAX* G-248A polymorphisms yielded the following results: GG, 81.53±13.23 months, GA, 67.17±10.47 months, and AA, 85.9±11.65 months. Thus, there were no statistically significant differences in survival times associated with different alleles in our pairwise analysis (p>0.05) (figures 5-7).

p-values: GG/AA: 0.866

GG/AG: 0.494

AA/AG: 0.368

Regarding our classification system (table I) the overall log-rank result was 12.466 with P=0.132, and we found no statistically significant differences between scoring categories. However, the pairwise analysis detected a statistically significant difference between the fourth (*BCL2* (AC)/*BAX* (AA)) and sixth category (*BCL2* (AC)/*BAX* (GG) (p=0.043).

## Discussion

The deregulation of pro- and anti-apoptotic mechanisms is well known to be closely related with the onset and rate of progression of malignant processes. Apoptosis is a transcriptionally controlled process in which BCL2 family members such as *BCL2*, *BCL-XL*, *BAK*, and *BAX* play a critical role in these pathways through their pro- or anti-apoptotic effects. The BCL2 and BAX proteins interact to maintain cell survival; hence, their quantitative ratio is crucial for apoptosis induction in normal or malignant cells. Several studies have shown that the modulation of *BCL2* expression affects cell susceptibility to apoptosis induced by anti-cancer drugs (11-14). Wojcik et al. showed that greater expression of *BCL2* mRNA was associated with a worse disease prognosis and a greater likelihood of relapse (15), .In the present study, AA allele frequency was related with elevated *BCL2* expression, as described before; the C allele interacts more powerfully with transcription factors and so by activating the P2 promoter region the activation of P1 promoter and so BCL2 expression reduces. In this case, A allele causes opposite of that process (7). 

High BCL2 level protects cells from apoptosis process by increasing the stability of mitochondrial outer membrane, thus, the intrinsic apoptotic pathways halts. Consequently the main target of most of natural and extrinsic anti-cancer materials, Apoptosis Induction, blocks. Therefore, the Kaplan-Meyer test showed that estimated survival was less than expected, in agreement with previous studies. Aref et al. found a relationship between *BCL2* expression and leukomogenesis in comparison to a healthy control group (16), and hypothesized that the AA allele occurs more frequently than other alleles. Our results confirmed this to be the case. Sahu and Das, found no correlation between *BCL2* expression and overall survival rate (17) and in the present study, our genetic analysis did not support this hypothesis. Campos et al. reported results similar to ours in adults with ALL (18); however, the relevance of these findings in the context of our results in a sample of pediatric patients with ALL is unclear. Despite these findings, Tzifi et al. claimed that successful induction therapy correlated with lower *BCL2* expression levels (19). Song et al. reported the similar data about esophageal cancer cells (20). Additionally, according to Campana et al. the level of *BCL2* expression influenced the sensitivity of leukemic cells to therapy because their high proliferation rate increased their susceptibility to toxic agents (21), Zhao et al. reported the similar results in NSCLC cells (22). In a study in Birmingham, UK, Starczynski et al. reported the frequencies of different polymorphisms as 85.1% for GG, 14.1% for GA and 0.8% for AA in their control group, compared to 77.4% for GG, 21.6% for GA and 1% for AA in patients with CLL (23). Previously, Hogarth et al. found that *BCL2* expression and the *BCL2*/*BAX* ratio had no prognostic significance in children with ALL, whereas a high expression of *BAX* at diagnosis was associated with a significant increase in the probability of relapse (24). Conversely, Sharifi et al. reported the favorable outcome in high *BCL2/BAX *ratio in Paclitaxel resistant breast cancer cell-line (25), and Jaafar et al. reported no significance between BCL2, BAX expression and breast tumor cells and angiogenesis of the tumor (26).

*BAX* plays a pivotal role in the mitochondrial pathway of apoptosis (27). In malignant cells, the induction of *BAX* initiates cell death, and *BAX* overexpression enhances apoptosis induced by chemotherapy (28). Then, it seems reasonable that a genetic susceptibility to greater *BAX* expression (GG allele) would be accompanied by low cell proliferation and less disease invasion, and be associated with a higher survival ratio. However, we found no statistically significant differences in survival time between patients with different *BAX* G-248A SNPs (figure 8). This is consistent with the findings reported by Anvari et al., who showed that the effects of *BAX* expression on other malignancies and the outcomes of different therapeutic methods were inconsistent (29). 

It is currently accepted that the *BAX* promoter contains the binding site for typical p53 tumor suppressor, as a result, the roll of p53 in the induction of apoptosis is due in part to increased BAX protein expression, since p53 induces apoptosis by activating the *BAX* gene (30). As a consequence, lower *BAX* levels in different tumors are not surprising since *BAX* is a transcriptional target of p53, which is mutated in most human cancers (30). According to Kirkin et. al. based on results from cell lines and animal models, negative control or inactivation of *BAX* and BAX-like death factors have been found in several human cancers (31). Decreased *BAX* expression has been reported in breast cancer (32), hepatocellular carcinomas (33) and chemoresistant B-cell chronic lymphocytic leukemia (CLL) (34). In some tumors, low *BAX* expression levels were found to be a negative prognostic factor for survival (35). Moreover, another study found that neither *BAX* expression levels nor *BAX*/*BCL2* ratio was significantly lower in patients who relapsed compared to the initial diagnosis (36), this ratio can be important, because Yu et al, reported the anti-hepatoma effect of curcumin based on increasing the expression of BAX and decreasing the BCL2 expression (37). In general, our scoring system indicates that despite the major roll of *BAX* in apoptosis induction, inheriting the SNPs that lead to higher expression of the BAX protein did not confer increased survival in our sample of patients with ALL (p>0.05). This paradoxical finding seems difficult to explain. Although *BAX* homodimers promote apoptosis, *BAX* and *BCL2* heterodimers prevent cell apoptosis (38), so equilibrium in *BCL2*/*BAX* and *BAX*/*BAX* ratios should be regarded as central to the molecular regulation of apoptotic pathways (38). The distribution of our patients in the different scoring categories can be explained on the basis of the relative allele frequencies. In the light of previous studies about the role of SNPs on *BAX* and *BCL2 *expression, we hypothesized that lower scores would be associated with a lower survival rate, a higher disease prevalence or a poor prognosis. This expected pattern can be seen in table I: lower scores of 1 to 4 are more frequent (38, 61.4%) than higher scores of 5 to 9 (24, 38.6%). 

According to reports published by the American Cancer Society and Cancer Research UK (39, 40), 88% to 95% of children with ALL are expected to survive for 5 years with standard anti-cancer therapy protocols. However, we found that the percentage of patients in Iran who are expected to survive for 5 years was much lower (40% to 65%). Although the efficacy of the treatment protocols seems to equal by comparison of the WBC count between control and ALL groups. Subsequently, additional studies will be needed to explain the differences in estimated survival.

We conclude that because of the role of pro- and anti-apoptotic members of the BCL2 family, our classification system should be evaluated with a multifactorial approach. Thus, more complex SNP evaluations and mathematical modeling will be needed to refine our system. Nevertheless, *BCL2* appears to be a reliable anti-apoptotic factor and its promoter region SNPs can be regarded as important factors to consider in any scoring system. In contrast, our findings for *BAX* SNPs were less conclusive because of the overlapping roles of some alleles, so our results should be considered with caution. These data are required to be confirmed with further studies consisting larger samples.

## Notes

**Funding:** This study was financially supported by Tabriz University of Medical Sciences, Deputy of Research and Technology (54/10286).
